# Supplementary material for: Structural basis for substrate gripping and translocation by the ClpB AAA+ disaggregase
Source: Nat Commun. 2019 Jun 3;10:2393. doi: 10.1038/s41467-019-10150-y (PMC6546751; doi:10.1038/s41467-019-10150-y)
Supplement: Supplementary file 1 — Supplementary Information [file 41467_2019_10150_MOESM1_ESM.pdf]

Supplementary Information

Structural basis for substrate gripping and translocation by the ClpB AAA+ disaggregase

Rizo, A.N., et al.

Supplementary Fig. 1

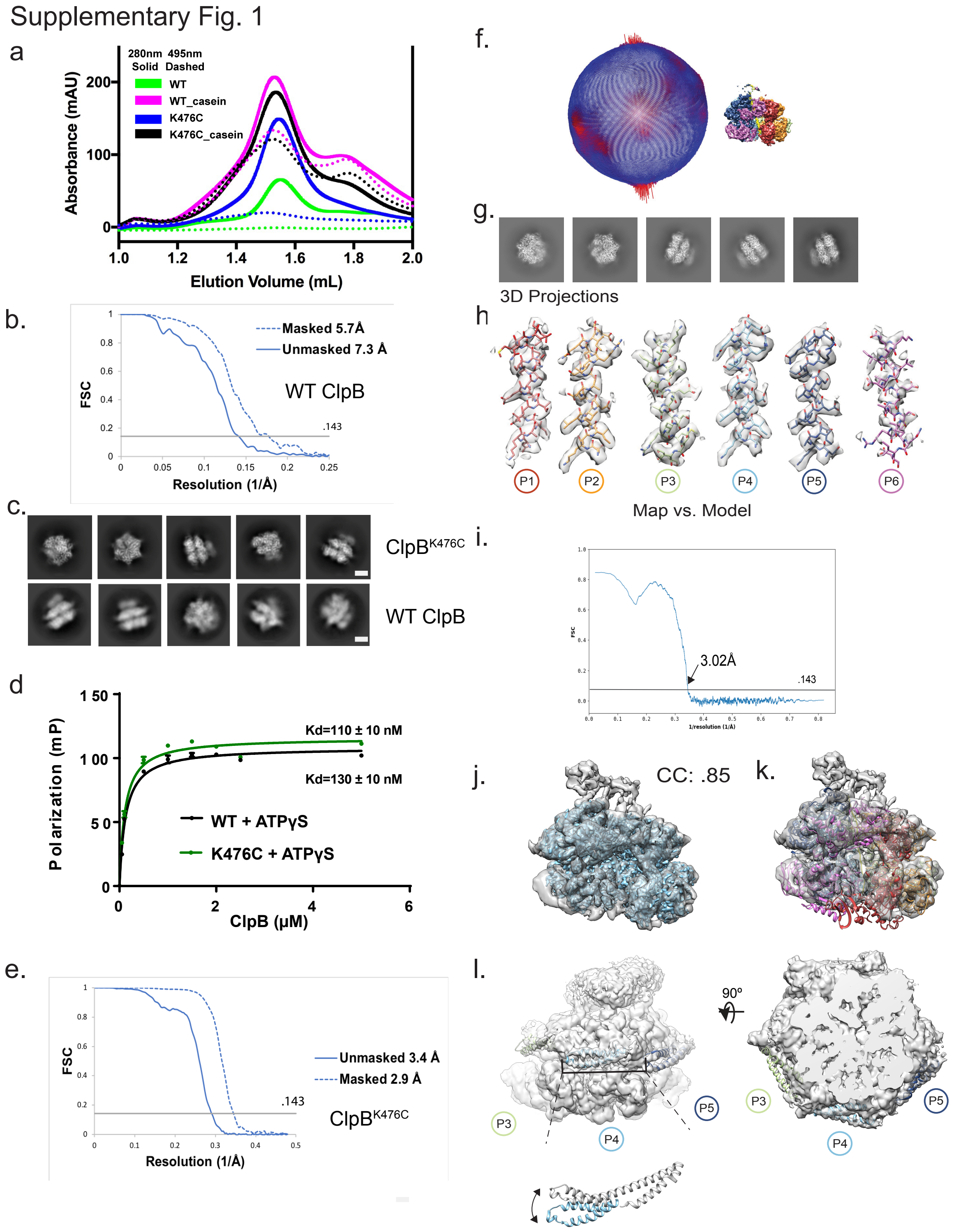

Supplementary Figure 1. ATPγS-ClpB^{K476C}:casein complex and analysis of cryo-EM data.
(a) Formation of the substrate-bound WT ClpB and ClpB^{K476C} complexes by size exclusion chromatography (SEC) following incubation with FITC-casein and ATPγS. Absorbance traces are shown for λ = 280 (solid) for protein, and λ = 495 (dash) for FITC. **(b)** Gold standard FSC-curve of the final map of WT ClpB:casein. **(c)** Reference-free 2D class averages of casein-bound WT and ClpB^{K476C} following SEC fractionation. The scale bar equals 50 Å. **(d)** Fluorescence polarization assay measuring FITC-casein binding with ClpB^{K476C} (green) and ClpB^{WT} (black) in the presence of ATPγS. **(e)** Gold standard FSC-curves of the final

ClpB^{K476C}:casein map using the total dataset following 2D classification. **(f)** Angular distribution of the particles for ClpB^{K476C}:casein reconstruction determined using RELION[1]. **(g)** Projections of ClpB^{K476C}:casein reconstruction showing top and side orientations that match reference-free averages in (c). **(h)** The cryo-EM map and atomic model of helix C3[2] that includes residues 388-406 is shown for each protomer. **(i)** The map vs. model FSC-curve following atomic modeling in PHENIX[3] **(j)** Alignment of WT ClpB:casein (grey) and ClpB^{K476C}:casein (blue) maps shown with the cross correlation (CC) value, calculated using UCSF Chimera[4]. **(k)** The WT ClpB:casein map docked with the molecular model determined from the ClpB^{K476C}:casein map. **(l)** Side (left) and top (right) views of ClpB^{K476C}:casein map from one class at an increased contour showing the MDs (helix 1 and helix 2) (pdb:5ofo) docked into the density for protomers P3-P7 in the “ATP-state” conformation, previously identified in Hsp104[5, 6]. Alignment to the MD conformation from ClpB-BAP^{DWB} (grey)[7] is shown below.

Supplementary Fig. 2

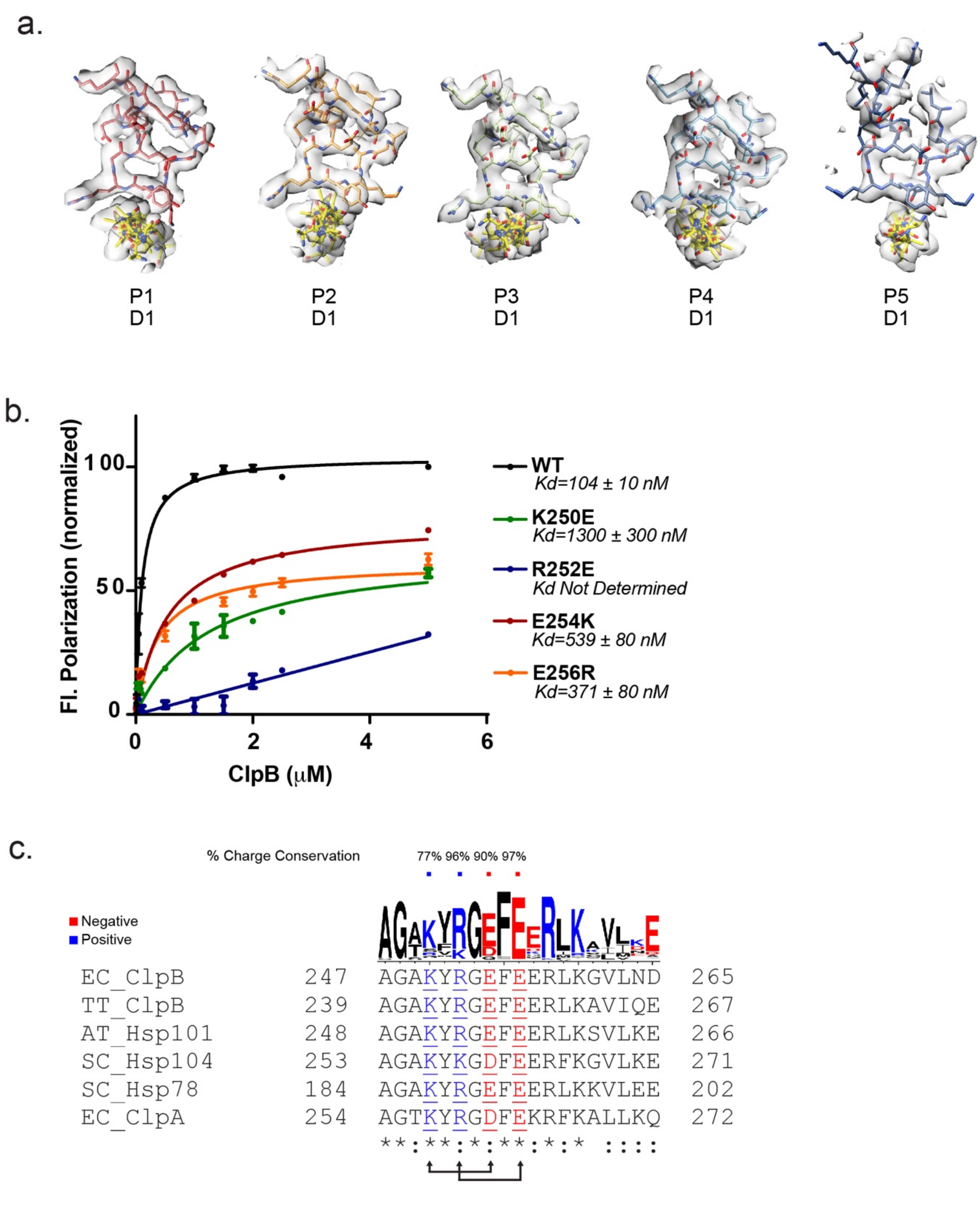

Supplementary Figure 2. Analysis of the D1 pore loops.
(a) Top view of the segmented map of the D1 loops from protomers 1-5, with residues (245-260) shown. **(b)** Fluorescence polarization assay measuring FITC-casein binding with $ClpB^{WT}$ (black), $ClpB^{K250E}$ (green), $ClpB^{R252E}$ (blue), $ClpB^{E254K}$ (red), or $ClpB^{E256R}$ (orange) in the presence of ATPγS. The Kds are indicated. **(c)** A multiple sequence alignment (MSA) with *E. coli* ClpB was performed against 6177 homologues using GREMLIN[8] (see materials and methods). The sequence logo for *E. coli* ClpB encompassing the D1 pore loop (residues 247–265) is shown, with alignment of *T. thermophilus* ClpB, *A. thaliana* Hsp101, *S. cerevisiae* Hsp104, *S. cerevisiae* Hsp78, and *E. coli* ClpA. Consensus symbols: identical (∗) and similar (**:**) conserved residues are indicated.

Supplemenatry Fig. 3

a.

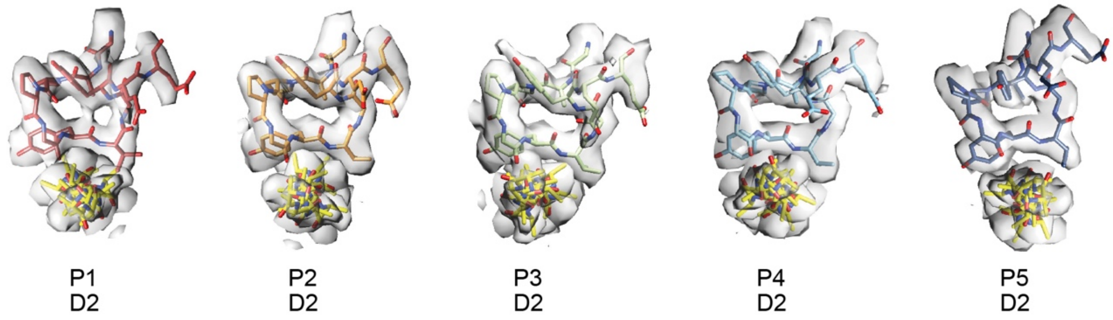

Supplementary Figure 3. Map+model views of the NBD2 pore loops.
Top view of the segmented map of the D2 loops from protomers 1-5, with residues (646-660) shown.

Supplementary Fig. 4

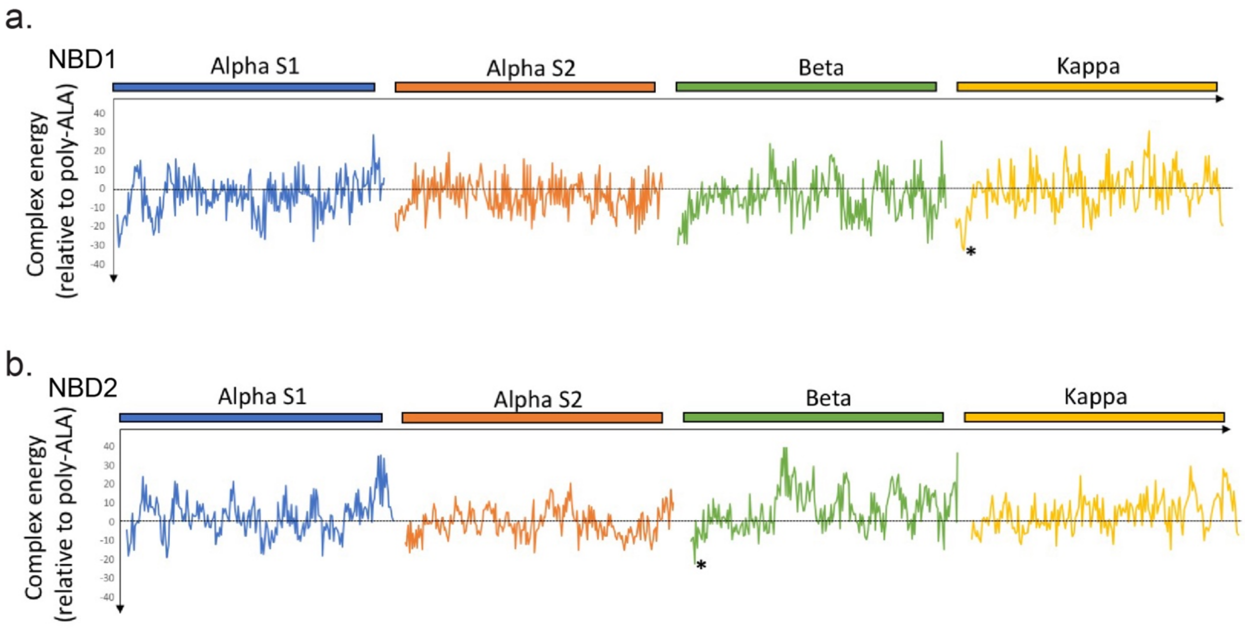

Supplementary Figure 4: Rosetta energies of overlapping casein peptides modeled for the NBD1 and NBD2. A sweep of the binding energies determined in Rosetta is plotted for the 1604 11-residue peptides from the N-termins to C-terminus of casein for the four isoforms in NBD1 **(a)** and NBD2 **(b)**. Energies are shown relatative to poly-Alanine. Peptides with the lowest energy that were modeled in the cryo-EM map and shown in Figure 4a and b are marked (*).

Supplementary Fig. 5

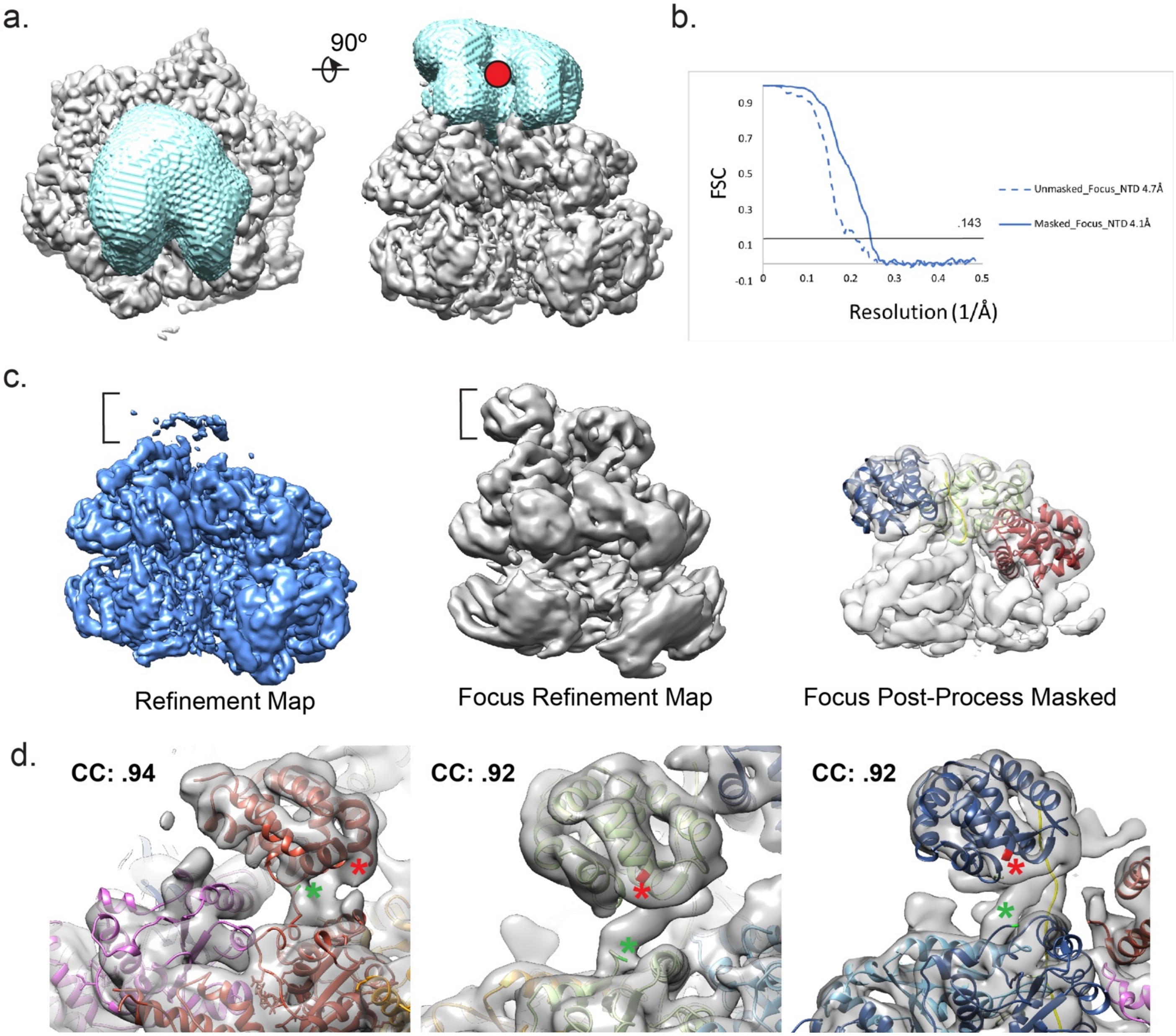

Supplementary Figure 5. Focus classification to improve resolution of NTDs.
(a) Map and mask used in the focus classification of the NTDs. Red dot represents the new center of mass in which the particles were adjusted to. **(b)** Gold standard FSC-curve of the post-process masked map of the NTD focus refinement map. Note, resolution from FSC appears overestimated based on map density, likely due to masking during refinement. **(c)** The original map (left, blue) compared to the focus classification map after refinement (middle, grey) and after applying the NTD mask during post-processing (right, docked with the NTD trimer), shown at a threshold of 4σ. NTDs indicated by the brackets. **(d)** Map of the NTD refinement classification docked with the model, pdb: 1KHY, chain A with the full ClpB^{K476C}:casein model. The N-terminus end of NBD1 (green *), residue161, and the C-terminus end of NTD (red *), residue142, are indicated. Connecting density adjacent these sites likely corresponds to NBD-NTD linker residues unresolved in previous structures. Cross-correlation values shown were calculated between the NTD focus classification map and model for each individual NTD using UCSF Chimera[4].

Supplementary Fig. 6

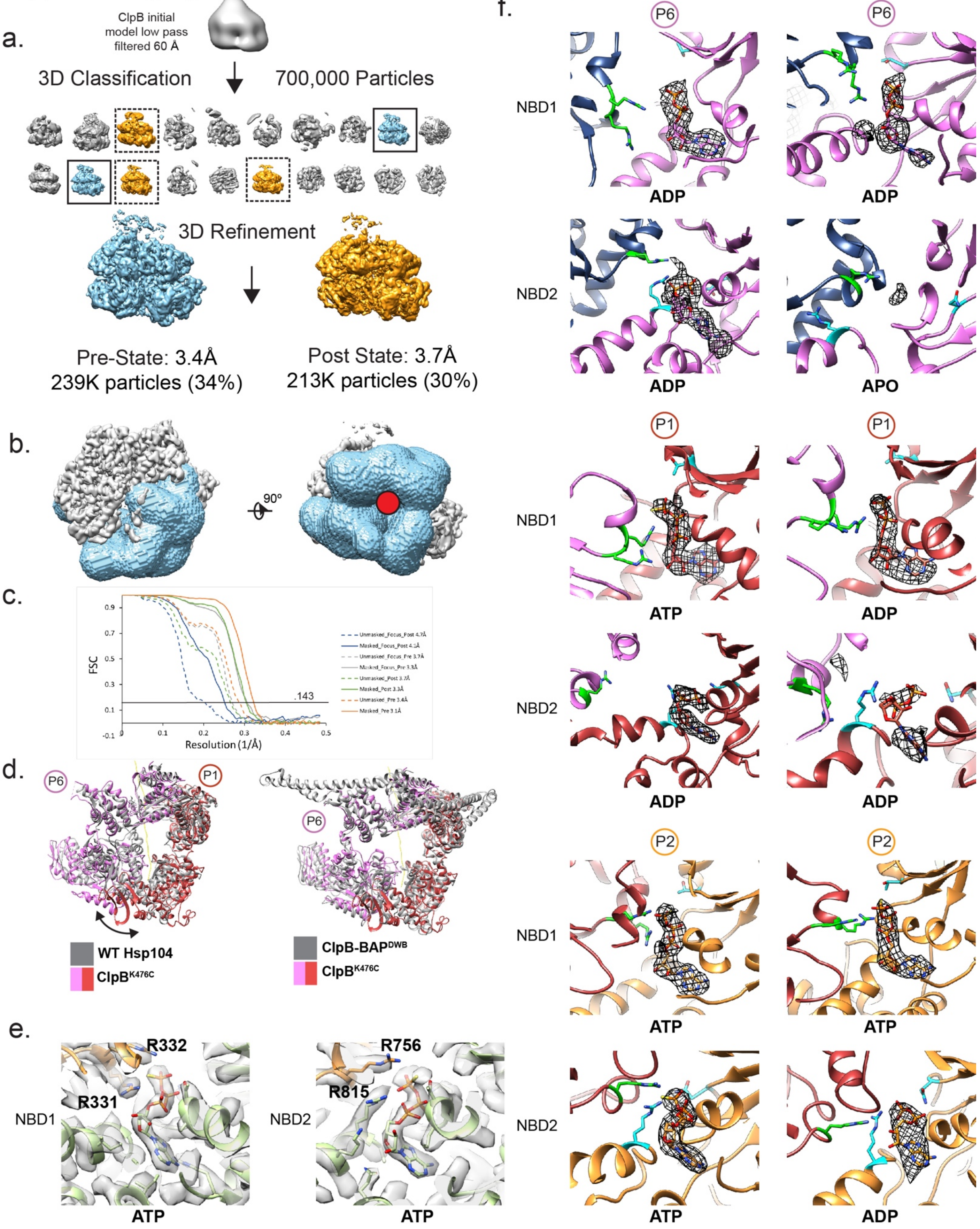

Supplementary Figure 6. Focus classification identifies two different conformations of the seam interface.

(a) 3D classification scheme that was used for the $ClpB^{K476C}$:casein dataset in identifying the two different states. The classes that were used in further refinement stages for each of the classes are identified by color, state 1 (blue) and state 2 (orange). Classes that were excluded from further refinement are indicated in grey. **(b)** Map and mask used in the focus classification of the two-flexible front protomers, P1 and P6. Red dot represents the new center of mass in which the particles were adjusted to. **(c)** Gold standard FSC-curves of the final maps of the Pre and Post states from 3D classification in (a) and focus refinements in (b). **(d)** Model of pre-state (colored) aligned with 5ofo (grey), with P1 and P6 shown. **(e)** Map plus model view of the nucleotide pockets of P3 NBD1 and NBD2 with Arg finger residues labeled, identifying the ATP state. The nucleotide pockets are identical for protomers P3-P5 in Pre and Post states, indicating an ATP state configuration for these protomers. **(f)** Views of the seam protomer nucleotide pockets for the NBD1 and NBD2 in Pre and Post states with difference map (experimental – apo) density shown in mesh at a threshold of ~2.0 σ to identify nucleotide occupancy. The Arg finger residues (331 and 332 for NBD1 and 756 for NBD2) are shown in green and sensor residues (315 for NBD1 and 719 and 815 for NBD2) are shown in cyan. The proposed nucleotide state is listed below and based on the difference map density for nucleotide and position of the Arg finger in the adjacent protomer.

Supplementary Table 1: Cryo-EM data collection, refinement and validation statistics. Information regarding data collection, processing, and atomic modeling parameters for the different structures.

	Pre-State EMDB 20004 PDB 6OAX	Post-State EMDB 20005 PDB 6OAY	Pre-State Focus EMDB 20049 PDB 6OG1	Post-State Focus EMDB 20050 PDB 6OG2	NTD-trimer Focus EMDB 20051 PDB 6OG3
**Data collection and processing**					
Magnification	48,450	48,450	48,450	48,450	48,450
Voltage (kV)	300	300	300	300	300
Electron exposure (e–/Å^2)	56 & 62	56 & 62	56 & 62	56 & 62	56 & 62
Defocus range (μm)	1.2-2	1.2-2	1.2-2	1.2-2	1.2-2
Pixel size (Å)	1.032	1.032	1.032	1.032	1.032
Symmetry imposed	C1	C1	C1	C1	C1
Initial particle images (no.)	778,521	712,910	712,910	712,910	712,910
Final particle images (no.)	712,910	213,059	221,083	91,601	93,588
Map resolution (Å)	2.9	3.3	3.3	4.1	4.1*
FSC threshold	.143	.143	.143	.143	.143
Map resolution range (Å)	2.3-4.0	2.3-4.3	n/a	n/a	n/a
**Refinement**					
Initial model used (PDB code)	5ofo	5ofo	5ofo	5ofo	1khy
Model resolution (Å)	3.0	3.6	3.2	3.9	4.3
FSC threshold	.143	.143	.143	.143	.143
Model resolution range (Å)					
Map sharpening *B* factor (Å^2)	-137.9	-105.1	-127.4	-180.4	-10.0
Model composition					
Non-hydrogen atom	27,831	27,389	9,195	9,003	1,609
Protein residues	3,500	3,434	1,152	1,128	402
Ligands	12	11	4	3	0
B factors (Å^2)					
Protein					
Ligand					
R.m.s. deviations					
Bond lengths (Å)	.009	.006	.009	.0007	.005
Bond angles (°)	1.089	1.069	1.207	1.175	1.260
Validation					
MolProbity score	2.27	2.23	2.6	2.6	.5
Clashscore	16	15.8	20.7	18.6	0
Poor rotamers (%)	.83	.94	1.45	1.57	0

*FSC resolution for EMDB 20051 is over estimated based on map density

Supplementary Table 2: Casein amino acid sequences from bovine (α-S1, α-S2, β, and κ isoforms) used in substrate modeling experiments.

**α-S1**
MKLLILTCLVAVALARPKHPIKHQGLPQEVLNENLLRFFVAPFPEVFGKEKVNELSKDIGSESTEDQAMEDIKQMEAESISSSEEIVPNSVEQKHIQKDDVPSERYLGYLEQLLRLKKYKVPQLEIVPNSAEERLHSMKEGIHAQQKEPMIGVNQELAYFYPELFRQFYQLDAYPSGAWYYVPLGTQYTDAPSFSDIPNPIGSENSGKTTMPLW
**α-S2**
MKFFIFTCLLAVALAKNTMEHVSSSEESIISQETYKQEKNMAINPSKENLCSTFCKEVVRNANEEEYSIGSSSEESAEVATEEVKITVDDKHYQKALNEINQFYQKFPQYLQYLYQGPIVLNPWDQVKRNAVPITPTLNREQLSTSEENSKKTVDMESTEVFTKKTKLTEEEKNRLNFLKKISQRYQKFALPQYLKTVYQHQKAMKPWIQPKTKVIPYVRYL
**β**
MKVLILACLVALALARELEELNVPGEIVESLSSSEESITRINKKIEKFQSEEQQQTEDELQDKIHPFAQTQSLVYPFPGPIPNSLPQNIPPLTQTPVVVPPFLQPEVMGVSKVKEAMAPKHKEMPFPKYPVEPFTESQSLTLTDVENLHLPLPLLQSWMHQPHQPLPPTVMFPPQSVLSLSQSKVLPVPQKAVPYPQRDMPIQAFLLYQEPVLGPVRGPFPIIV
**κ**
MMKSFFLVVTILALTLPFLGAQEQNQEQPIRCEKDERFFSDKIAKYIPIQYVLSRYPSYGLNYYQQKPVALINNQFLPYPYYAKPAAVRSPAQILQWQVLSNTVPAKSCQAQPTTMARHPHPHLSFMAIPPKKNQDKTEIPTINTIASGEPTSTPTIEAVESTVATLEASPEVIESPPEINTVQVTSTAV

Supplementary Table 3: Casein substrate peptide modeling.
Sequence and binding energy change for the five lowest (most favorable) and five highest (least favorable) scoring casein peptides.

**NBD1**		
	**Casein Sequence**	**Energy Delta**
Favorable	VVTILALTLPF	-32.9
	LVVTILALTLP	-31.5
	LLILTCLVAVA	-30.8
	KVLILACLVAL	-28.8
	LVALALARELE	-28.6
Unfavorable	FPGPIPNSLPQ	21.8
	YPFPGPIPNSL	24.5
	EPVLGPVRGPF	25.9
	SDIPNPIGSEN	28.5
	PPKKNQDKTEI	30.0
**NBD2**		
	**Casein Sequence**	**Energy Delta**
Favorable	ILACLVALALA	-21.7
	NENLLRFFVAP	-18.8
	LLILTCLVAVA	-18.0
	MIGVNQELAYF	-17.7
	RYLGYLEQLLR	-17.1
Unfavorable	PSFSDIPNPIG	35.2
	FSDIPNPIGSE	35.5
	LGPVRGPFPII	37.2
	PFPGPIPNSLP	42.1
	PGPIPNSLPQN	44.1

Supplementary Table 4
Primer-sets used to make ClpB variants via Quick-change reaction.

Primer set #	Primer name	Primer sequences (sense and anti-sense)
1	ClpB K250E	5'-aaactcaccgcgatactccgccccagccaccag-3'<br>5'-ctggtggctggggcggagtatcgcggtgagttt-3'
2	ClpB R252E	5'-tcttcaaactcaccctcatatttcgccccagccaccagc-3'<br>5'-gctggtggctggggcgaaatatgagggtgagtttgaaga-3'
3	ClpB E254K	5'-aacgttcttcaaacttaccgcgatatttcgcccca-3'<br>5'-tggggcgaaatatcgcggtaagtttgaagaacgtt-3'
4	ClpB E256R	5'-cttttaaacgttctctaaactcaccgcgatatttcgccccag-3'<br>5'-ctggggcgaaatatcgcggtgagtttagagaacgtttaaaag-3'
5	ClpB E639K	5'-gagacaccgagtgtttcttcataaactcggacatatc-3'<br>5'-gatatgtccgagtttatgaagaaacactcggtgtctc-3'
6	ClpB K640E	5'-ccaaacgagacaccgagtgctcctccataaactcggacata-3'<br>5'-tatgtccgagtttatggaggagcactcggtgtctcgtttgg-3'
7	ClpB H641E	5'-aacgagacaccgactctttctccataaactcggacatatcg-3'<br>5'-cgatatgtccgagtttatggagaaagagtcggtgtctcgtt-3'

All primers are ordered from IDT® (integrated DNA technologies).

Supplementary References

1. Scheres SH. RELION: implementation of a Bayesian approach to cryo-EM structure determination. *J Struct Biol* **180**, 519-530 (2012).

2. Lee S*, et al.* The structure of ClpB: a molecular chaperone that rescues proteins from an aggregated state. *Cell* **115**, 229-240 (2003).

3. Adams PD*, et al.* PHENIX: a comprehensive Python-based system for macromolecular structure solution. *Acta Crystallogr D Biol Crystallogr* **66**, 213-221 (2010).

4. Pettersen EF*, et al.* UCSF Chimera--a visualization system for exploratory research and analysis. *J Comput Chem* **25**, 1605-1612 (2004).

5. Gates SN*, et al.* Ratchet-like polypeptide translocation mechanism of the AAA+ disaggregase Hsp104. *Science* **357**, 273-279 (2017).

6. Yokom AL*, et al.* Spiral architecture of the Hsp104 disaggregase reveals the basis for polypeptide translocation. *Nat Struct Mol Biol* **23**, 830-837 (2016).

7. Deville C*, et al.* Structural pathway of regulated substrate transfer and threading through an Hsp100 disaggregase. *Sci Adv* **3**, e1701726 (2017).

8. Ovchinnikov S, Kamisetty H, Baker D. Robust and accurate prediction of residue-residue interactions across protein interfaces using evolutionary information. *Elife* **3**, e02030 (2014).
