# Supplementary material for: Structural basis for substrate gripping and translocation by the ClpB AAA+ disaggregase
Source: Nat Commun. 2019 Jun 3;10:2393. doi: 10.1038/s41467-019-10150-y (PMC6546751; doi:10.1038/s41467-019-10150-y)
Supplement: Supplementary file 3 — Description of Additional Supplementary Files [file 41467_2019_10150_MOESM3_ESM.pdf]

Description of Additional Supplementary Files

File Name: Supplementary Movie 1

Description: Pre and Post state conformational changes. Movie showing a morph between the Pre and Post state conformations of the seam interface protomers P1 and P6.

File Name: Supplementary Movie 2

Description: Protomer conformational changes in the hexamer. Movie showing a morph between the different conformational changes of the protomers we attribute to the ATP hydrolysis cycle of the NBD1 and NBD2 during substrate translocation. The conformational changes are based on alignment to the NBD1 small subdomains of the individual protomers in the Pre and Post states.
